# Tableau de cécité bilatérale à fond d’œil normal révélant un anévrisme non rompu de l'artère cérébrale communicante antérieure

**DOI:** 10.11604/pamj.2014.17.178.3709

**Published:** 2014-03-07

**Authors:** Abdellah Alaoui Ismaili, Meriem Abdellaoui, Zineb Khrifi, Noureddine Eddassi, Ourda Nejjari, Idriss Benatiya Andaloussi, Hicham Tahri

**Affiliations:** 1 Service d'Ophtalmologie CHU Hassan II, Fès, Maroc

**Keywords:** Cécité, fond d’œil, anévrisme, artère cérébrale, blindness, fundus, aneurism, cerebral artery

## Abstract

Les anévrysmes intracrâniens non rompu (AICNR) se manifestent le plus souvent par des tableaux cliniques non spécifiques (céphalées, accidents vasculaires ischémiques cérébraux, paralysies des nerfs crâniens) et sont généralement découverts à l'occasion d'imagerie cérébrale pour d'autres raisons. Une cécité bilatérale révélant AICNR de l'artère communicante antérieure est unique dans la littérature. Il s'agit d'un homme de 48 ans sans antécédents médicaux notables qui consulte aux urgences ophtalmologiques pour baisse de l'acuité visuelle rapidement évolutive en une semaine aboutissant à une cécité bilatérale. L'examen ophtalmologique objective une acuité visuelle réduite à la présence d'une perception lumineuse bilatérale avec un bon réflexe photomoteur et le reste de l'examen ophtalmologique qui est strictement normal. Un examen général réalisé en urgence trouve une tension artérielle à 20/14mmHG. L'imagerie cérébrale notamment une tomodensitométrie cérébrale complétée par une imagerie en résonnance magnétique objective un aspect spectaculaire d'un grand anévrisme de l'artère cérébrale communicante antérieure partiellement thrombosé responsable d'une compression isolée du chiasma optique. Après un équilibre de l'hypertension artérielle le patient a été adressé en neurochirurgie pour une thérapeutique en urgence. Un anévrisme est toujours la conséquence d'une modification structurale de la paroi artérielle. Dans leur forme commune, les anévrismes développés sur les artères intracrâniennes sont sacciformes. Ses étiologies sont nombreuses et variées. L’évolution des anévrysmes intracrâniens se fait vers une augmentation de leur volume sous l'influence de facteurs hémodynamiques qui conduit à une fragilisation de leur paroi. Une rupture de l'anévrisme est le mode de découverte le plus fréquent et aussi le plus dramatique. Toute baisse d'acuité visuelle à fond d'oeil normal doit imposer sans retard une imagerie orbito-cérébrale.

## Introduction

Les anévrysmes intracrâniens non rompu (AICNR) sont rarement symptomatiques et sont le plus souvent découverts à l'occasion d'imagerie cérébrale pour d'autres raisons ou par des symptômes vagues comme des céphalées, des étourdissements ou une paralysie oculomotrice. Une cécité bilatérale révélant AICNR de l'artère communicante antérieure est unique dans la littérature. Les anévrysmes sont dus à une modification structurale de la paroi artérielle d'origine diverse. Sur un mode de révélation dramatique et sous l'influence des facteurs hémodynamiques les anévrismes intracrâniens évoluent vers la rupture par une augmentation du volume et fragilisation de leur paroi.

## Patient et observation

**Figure 1:**
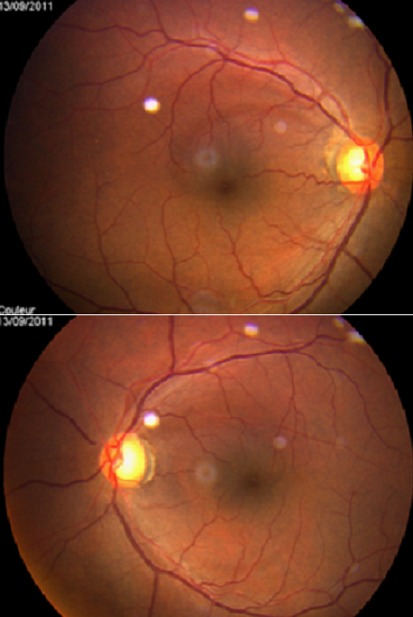
Aspect normal du fond d’œil

**Figure 2:**
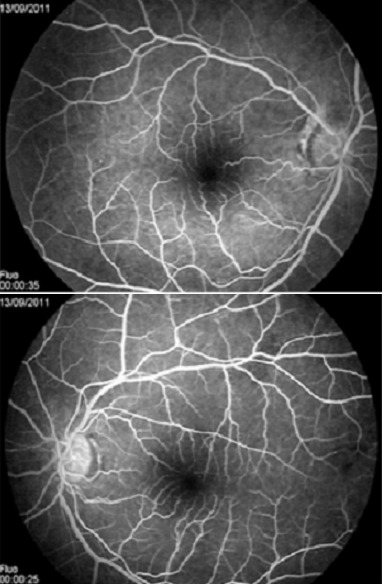
Aspect au temps précoce du fond d’œil après injection de fluorescéine

**Figure 3:**
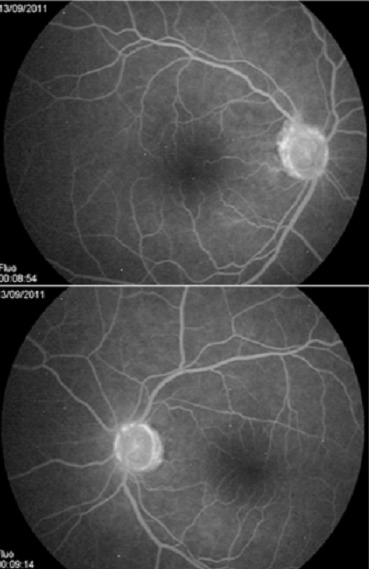
Aspect au temps tardif du fond d’œil après injection de fluorescéine

**Figure 4:**
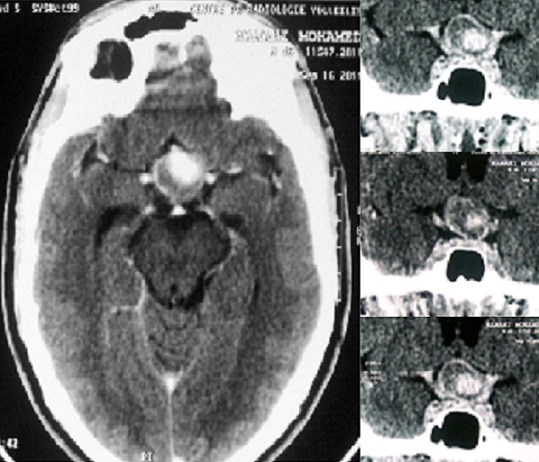
TDM cérébrale en coupe axiale et coronales montre une lésions arrondie antéhypophysaire hétéro-dense se rehaussant après injection de produit de contraste iodé et responsable d'une compression opto-chiasmatique centrale

**Figure 5:**
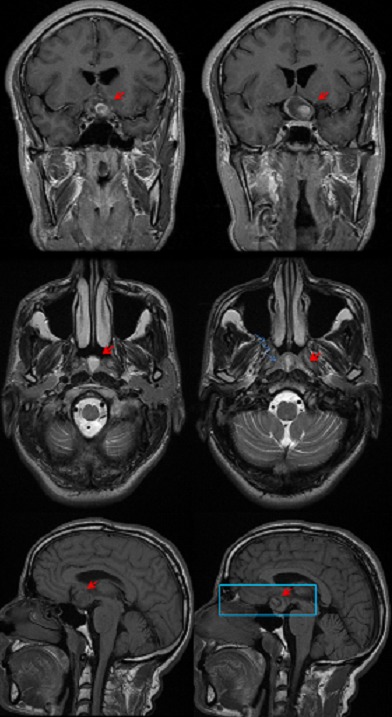
IRM cérébrale en coupe frontales, axiales et sagittales confirmant l'anévrysme thrombosé non rompu de l'artère cérébrale communicante antérieure et comprimant le carrefour opto-chiasmatique

Il s'agit d'un patient de 48 ans sans antécédents médicaux notables qui consulte aux urgences ophtalmologiques pour baisse de l'acuité visuelle rapidement évolutive en une semaine aboutissant à une cécité bilatérale. La pratique d'un examen ophtalmologique retrouve une acuité visuelle limitée à la présence d'une perception lumineuse aux deux yeux avec un réflexe photo-moteur direct et consensuel conservés, le reste de l'examen ophtalmologique est normal notamment du fond d'oeil (Figure 1). Devant ce tableau, en restons systématiques, l'examen des différents appareils est sans particularité à l'exception d'une tension artérielle 20/14mmHG. L'angiographie rétinienne à la fluorescéine ne montre pas de diffusion papillaire aux temps tardive ou des signes de vascularite (Figure 2, Figure 3). La TDM cérébrale en coupe axiale et coronales montrait une lésions arrondie antéhypophysaire hétéro-dense se rehaussant après injection de produit de contraste iodé et responsable d'une compression opto-chiasmatique centrale (Figure 4). L'IRM cérébrale en coupe frontales, axiales et sagittales confirmait l'anévrysme thrombosé non rompu de l'artère cérébrale communicante antérieure et comprimant le carrefour opto-chiasmatique (Figure 5). Rapidement le patient a été envoyé en neurochirurgie pour un clippage de l'anévrysme, malheureusement l’évolution a été marquée par une mort subite probablement due à la rupture de l'anévrisme secondaire à une hémorragie méningée.

## Discussion

Les principales manifestations cliniques des AICNR sont les céphalées (36%), les accidents vasculaires ischémiques cérébraux (17,6%) et les paralysies des nerfs crâniens (15,4%). Jamais un mode de révélation par une cécité bilatérale n'a été décrit [1, 2]. Les anévrysmes intracrâniens (AIC) rompus sont de loin la cause la plus fréquente des hémorragies méningées non traumatiques, c'est une urgence neurologique avec des conséquences potentiellement dévastatrices [3]. L'hypertension artérielle, le tabagisme et la consommation excessive d'alcool sont reconnus comme étant des facteurs de risque de développement des AIC. Le facteur de risque principal chez notre patient été une hypertension artérielle sévère.

Les causes de l'anévrisme cérébral et du processus de formation, de croissance et de rupture de l'anévrisme, sont mal connus. Des atteintes structurales de la limitante élastique interne et de la musculeuse des artères cérébrales basales, associées à des facteurs hémodynamiques, sont présumées être responsables de ces excroissances vasculaires. Dans leur forme commune, les anévrismes développés sur les artères intracrâniennes sont sacciformes, comme chez notre patient, et leur évolution se fait vers une augmentation de leur volume sous l'influence de facteurs hémodynamiques qui conduit à une fragilisation de leur paroi. Une rupture de l'anévrisme est le mode de découverte le plus fréquent et aussi le plus dramatique avec presque 50% de mortalité.

Les AIC se situent le plus souvent sur la bifurcation vasculaire du polygone de Willis essentiellement sur l'artère communicante antérieure, puis la cérébrale moyenne et la communicante postérieure et les autres. Ces anévrismes sont parfois multiples. Les AIC non rompus touche jusqu’à 6% de la population générale. La plupart des personnes atteintes restent asymptomatiques. Un dépistage est controversé à cause de la morbidité et la mortalité importantes associées à leur traitement chirurgical. L’évolution spontanée est aussi fatale chez nombreux malades liée au risque imminent d'une rupture de l'anévrysme. La neuroradiologie basée sur la tomodensitométrie, l'angiographie par tomodensitométrie, l'angiographie par résonance magnétique et l'angiographie par soustraction numérique est indispensable pour le diagnostic des AIC [4].

La prise en charge se base sur les techniques microchirurgicales ou endo-vasculaire en fonction de la localisation, de la taille et de la forme de l'anévrisme et aussi de la présence de maladies concomitantes de l’âge et l’état neurologique du patient. Les nouveaux matériaux et technologies endovasculaires, ainsi que les angiographies intra-opératoires ou les interventions combinées (hybrides), confèrent un potentiel très prometteur au traitement des anévrismes cérébraux.

## Conclusion

La cécité bilatérale est un mode rare de révélation d'anévrysme non rompu de la communicante antérieure. L'imagerie orbito-cérébrale est indispensable devant une baisse d'acuité visuelle avec examen ophtalmologique normal [5].
